# Supplementary material for: Microbiome of Invasive Tick Species Haemaphysalis longicornis in North Carolina, USA
Source: Insects. 2024 Feb 24;15(3):153. doi: 10.3390/insects15030153 (PMC10970973; doi:10.3390/insects15030153)
Supplement: Supplementary file 1 [file insects-15-00153-s001.zip › insects-2869750-supplementary.pdf]

Table S1. Summary of this table includes the location in which they were collected and the collection type

Location (County)	Stage/Sex	Year	Collection Type
Ashe	Female	2021	Cattle
Buncombe	Nymph	2019 -2020	Dragging
Catawba	Nymph	2021	Dragging
Surry	Female/ Larve/ Nymph	2019-2020	Dragging
Surry	Female	2020	Calf
Madison	Nymph	2019	Dragging

Table S2. Genus-level bacterial taxa with differential abundances between ALT larvae, nymphs, and adults; determined by ANCOM

Genus	ANCOM W-Value	Average (SE) Abundance (%)		
		Larvae	Nymphs	Adults
Coxiella	495	8.90 (1.76)	72.27 (4.28)	47.45 (6.19)
Staphylococcus	481	6.39 (1.00)	0.48 (0.30)	8.22 (3.36)
Sphingobium	455	5.59 (1.02)	1.11 (0.37)	0.91 (0.37)
Dietziaceae *unclassified genus*	451	3.53 (0.89)	0.24 (0.24)	0.54 (0.11)
Corynebacterium	457	1.46 (0.27)	0.15 (0.08)	1.40 (0.59)
Propionibacterium	478	2.06 (1.01)	0.26 (0.10)	0.04 (0.01)
Rhizobium	473	1.37 (0.24)	0.17 (0.05)	0.56 (0.14)
Clostridium	458	0.006 (0.004)	0.007 (0.006)	0.712 (0.209)
Dermabacteraceae *unclassified genus*	446	0.437 (0.099)	0.025 (0.025)	0.168 (0.040)

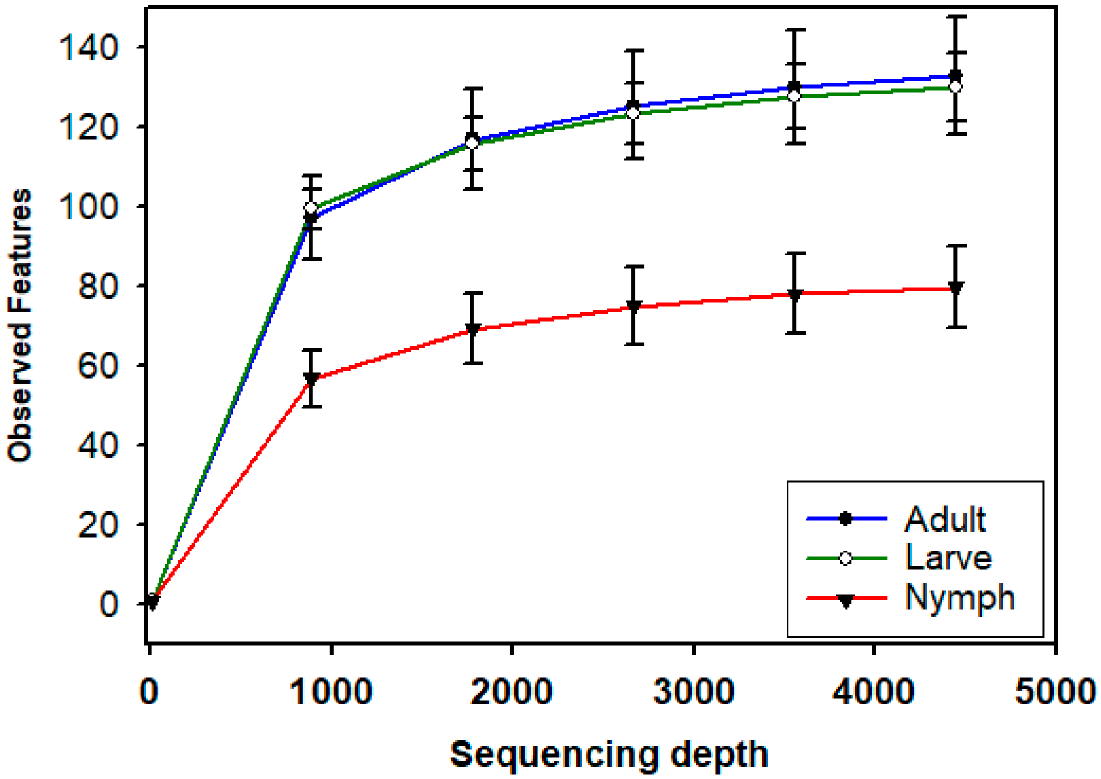

Figure S1. Rarefaction curves of observed features in larve, nymph and adult ticks. Error bars represent the standard error of the mean.

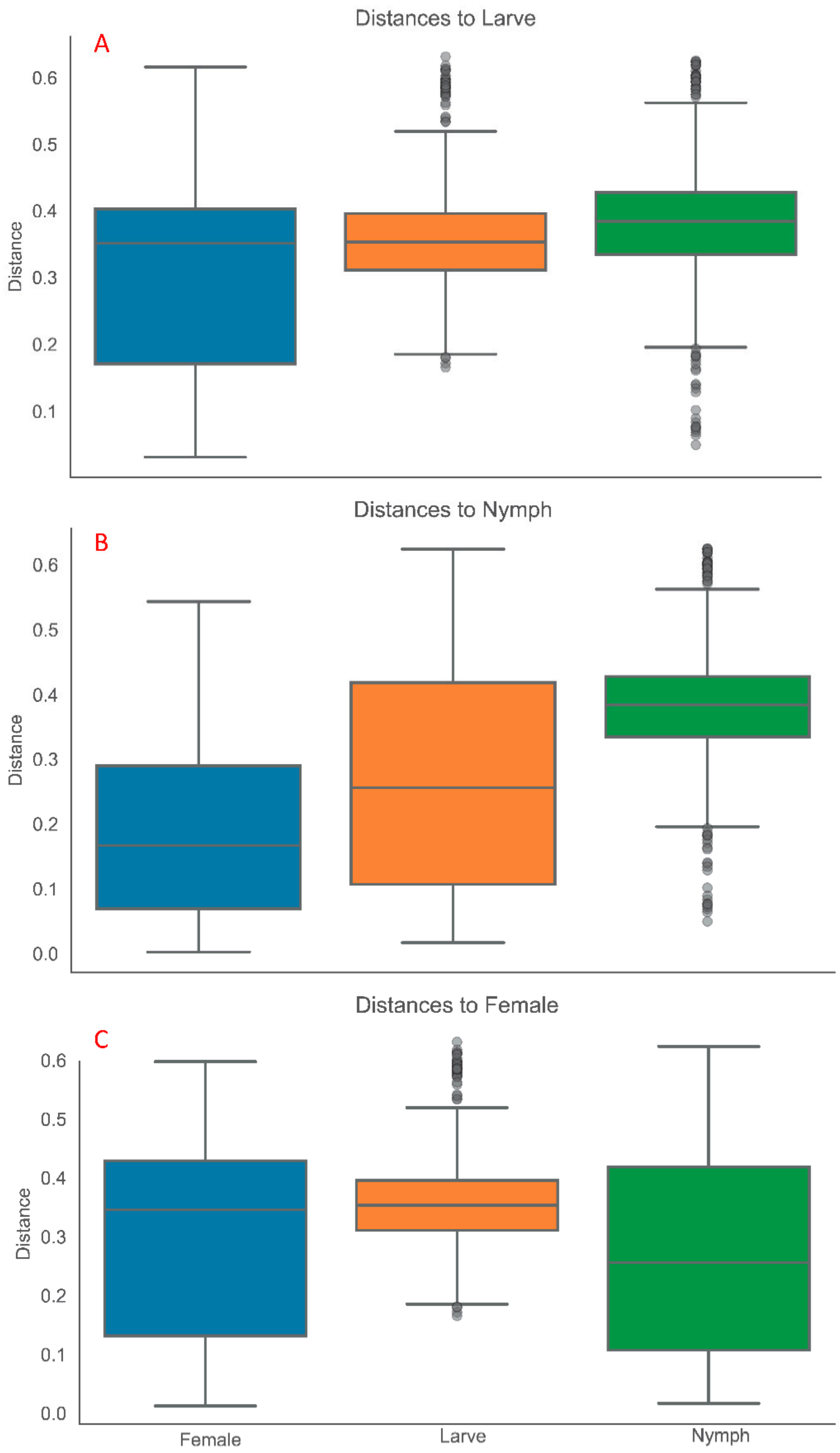

Figure S2. Beta diversity (box plot) measures of the on bacterial community composition (weighted UniFrac) to different stages.

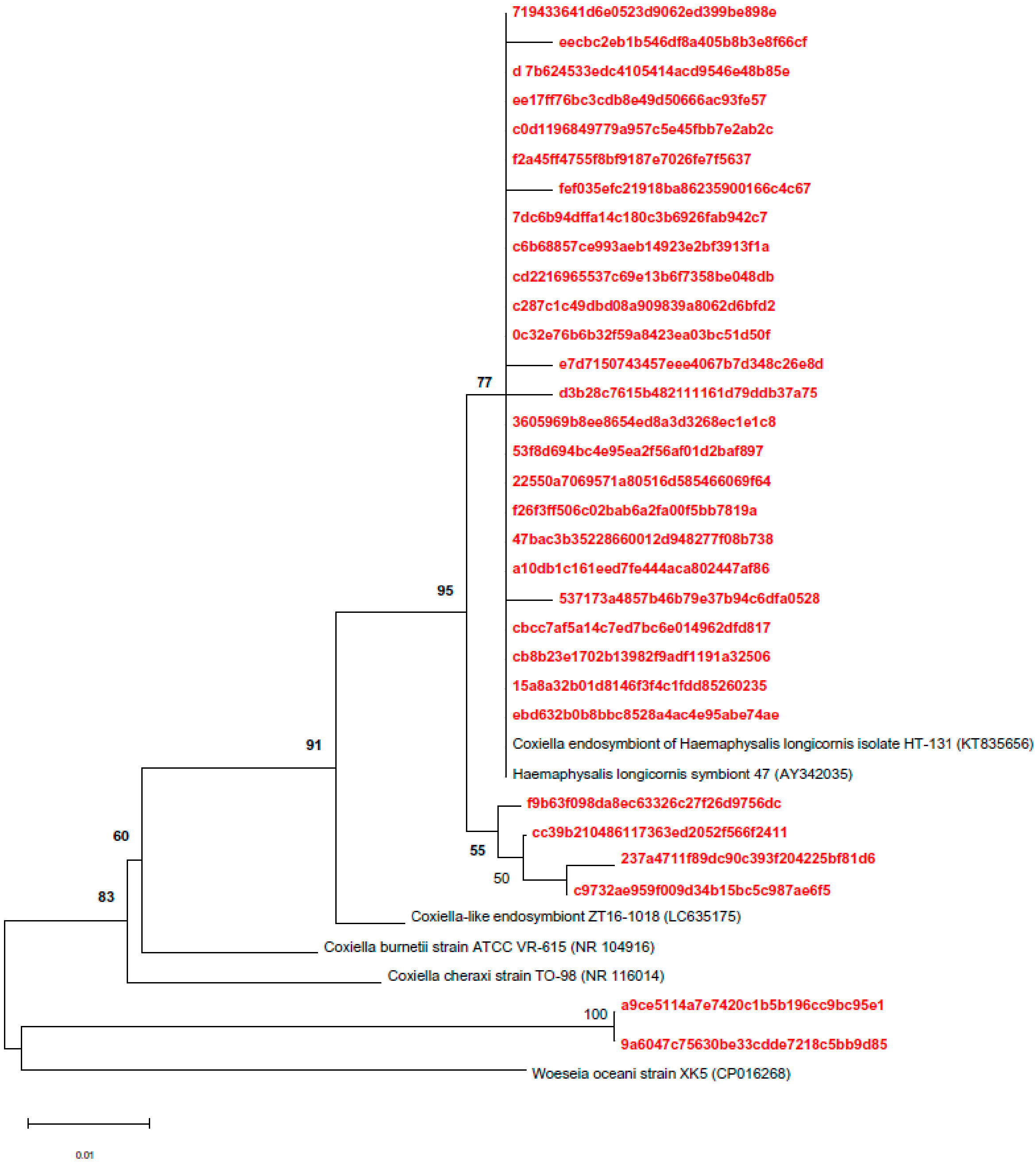

Figure S3. Phylogenetic analysis of *Coxiella* ASVs identified in this study (in red text) and closest related species and constructed by the neighbor-joining (NJ) method based on partial 16S rRNA gene. Bootstrap values above 50 % based on 1000 replications are indicated at the branches. *Woeseia oceani* strain XK5 (CP016268) used as outgroup.

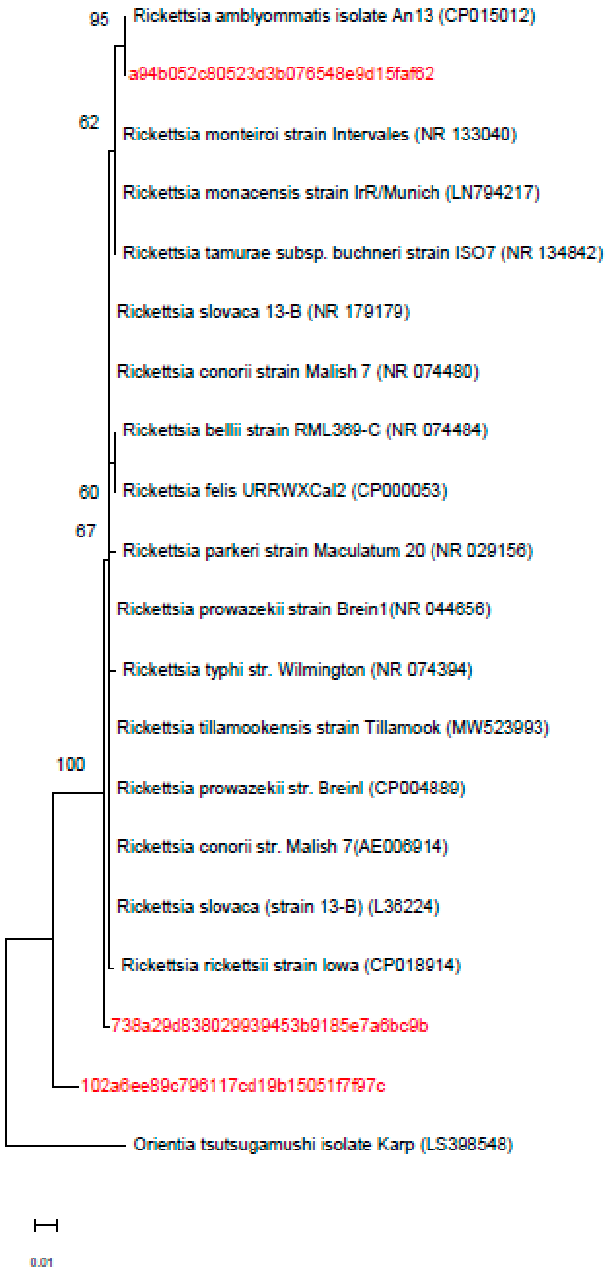

Figure S4. Phylogenetic analysis of *Rickettsia* ASVs identified in this study (in red text) and closest related species and constructed by the neighbor-joining (NJ) method based on 16S rRNA gene. Bootstrap values above 50 % based on 1000 replications are indicated at the branches. *Orientia tsutsugmashi* isolate Karp (LS398548) used as outgroup.

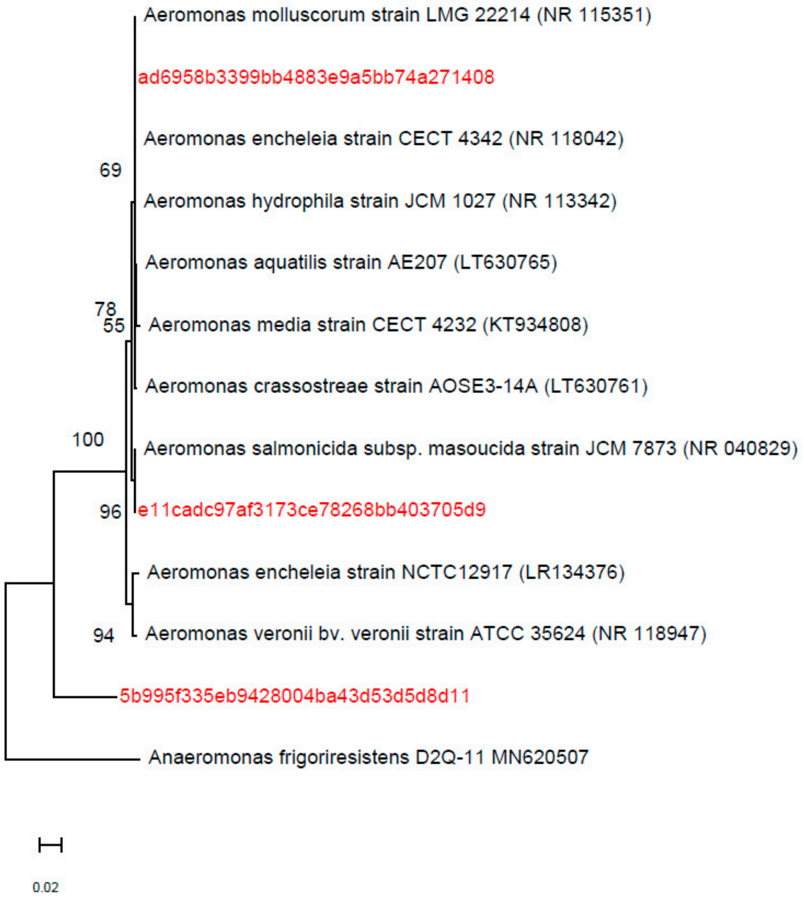

Figure S5. Phylogenetic analysis of *Aeromonas* ASVs identified in this study (in red text) and closest related species and tree constructed by the neighbor-joining (NJ) method based on 16S rRNA gene. Bootstrap values above 50 % based on 1000 replications are indicated at the branches. *Anaeromonas frigoriresistens* D2Q-11 MN620507 used as outgroup.
